# Same‐day antiretroviral therapy is associated with increased loss to follow‐up in South African public health facilities: a prospective cohort study of patients diagnosed with HIV

**DOI:** 10.1002/jia2.25529

**Published:** 2020-06-08

**Authors:** Dvora Joseph Davey, Kathleen Kehoe, Claire Serrao, Marlien Prins, Ntokozo Mkhize, Khanyo Hlophe, Senate Sejake, Todd Malone

**Affiliations:** ^1^ Department of Epidemiology Fielding School of Public Health University of California Los Angeles CA USA; ^2^ Division of Epidemiology and Biostatistics School of Public Health and Family Medicine University of Cape Town South Africa; ^3^ BroadReach Healthcare Cape Town South Africa; ^4^ Department of Health Durban South Africa

**Keywords:** antiretroviral therapy, ART, HIV, same‐day, retention, lost to follow‐up, South Africa

## Abstract

**Introduction:**

South Africa introduced Universal Test and Treat in 2016 including antiretroviral therapy (ART) initiation on the same‐day as HIV diagnosis. Our study sought to evaluate the impact of same‐day ART initiation on loss to follow‐up (LTFU) and mortality comparing with patients who initiated ART after their HIV diagnosis.

**Methods:**

We conducted a file review of patients with a HIV diagnosis and ART start date on file between September 2016 and May 2018 in six high HIV burden districts. Our primary outcome was LTFU (>90 days from the last clinical visit or drug pick‐up until database closure 31 July 2018). The secondary outcome was mortality after ART initiation. Time to outcome was assessed comparing same‐day vs. one to seven, eight to twenty‐one and ≥ twenty‐two days to ART initiation using Kaplan‐Meier estimators stratified by sex. We investigated predictors using univariate and multivariable Cox proportional hazards models, adjusting for *a priori* characteristics.

**Results:**

Overall, 92,609 ART patients contributed 43,922 person‐years from ART initiation, with a median follow‐up time of 246 days (IQR = 112 to 455). Of these patients, 33,399 (36%) initiated ART on the same‐day as their HIV diagnosis date and had a median follow‐up time of 174 days (IQR = 85 to 349). Same‐day patients were predominantly non‐pregnant females (56%) and aged 25 to 34 years (40%). Same‐day ART initiation increased from 2.8% in September 2016 to 7.1% in April 2018. In same‐day patients, 33% (n = 11,114) were classified as LTFU with a median time of 55 days (IQR = 1 to 185), compared to 371 mean days (IQR = 161 to 560) in patients who initiated ≥22 days after diagnosis. A similar proportion of LTFU was observed for patients who initiated later: 31% 1 to 21 day and 33% ≥22 day. Same‐day ART patients had an increased risk of LTFU vs. ≥1 day (adjusted hazard ratio (aHR) = 1.28, 95% CI = 1.24 to 1.33) adjusting for covariates. Although all‐cause mortality was slightly lower in same‐day patients (0.9%) vs. >1 day (1.4%; aHR = 0.87, 95% CI = 0.72 to 1.05) adjusting for covariates. Men had highest risk of mortality and LTFU.

**Conclusions:**

Same‐day ART increased the risk of LTFU, but same‐day patients experienced slightly lower mortality. Same‐day patients may require additional counselling and interventions to improve retention. Additional research is needed on targeted interventions, including differentiated care, to reduce LTFU in patients initiating ART same‐day.

## 1 Introduction

Innovative, cost‐effective and scalable approaches are needed to meet UNAIDS’ ambitious 90‐90‐90 goals: 90% of people living with HIV (PLHIV) know their serostatus, 90% of those are on sustained antiretroviral therapy (ART) and 90% of those are virally suppressed [1]. To reach those targets, South Africa adopted a universal test and treat policy in September 2016, which included endorsement of same‐day ART for clinically stable patients [2]. In South Africa, the country with the largest HIV epidemic and ART programme in the world, an estimated 91% of people living with HIV (PLHIV) know their HIV status. Of those, 68% were on ART, and 83% had achieved viral suppression as of June 2019 [3]. Targeted interventions are needed to improve linkage to ART and retention in care, especially in men who have poorer treatment uptake, retention and viral suppression compared to women living with HIV [4, 5, 6].

Attrition during the period from HIV testing to ART initiation remains high in sub‐Saharan Africa [7, 8]. PLHIV have cited that a common barrier to starting ART are frequent clinical visits and long waiting times involved [7]. Initiating ART on the same‐day as the patient’s HIV diagnosis is an innovation that is feasible in both clinical and community‐based settings [7, 9]. Studies have found improved linkage to ART leads to greater viral suppression rates [10, 11, 12]. A recent South African study demonstrated that initiating ART at the patient’s first clinic visit was cost‐effective, even with point‐of‐care laboratory tests included, compared to the standard of care which required several additional clinic visits [13]. In the United States, a recent study found that loss to follow‐up (LTFU) was similar between same‐day initiators and standard of care, and viral suppression was achieved more quickly in the intervention patients treated in the same clinic [14]. In a recent systematic review of randomized trials, same‐day ART increased likelihood of starting ART within 90‐days, viral suppression and retention in care at 12 months [15]. In observational studies, any ART initiation within three months was higher in rapid ART group; however, there was a non‐significant trend toward decreased retention in same‐day ART patients [15]. A recent South African clinical file review found that same day initiation was associated with poorer retention when compared to later initiation [16].

The World Health Organization (WHO) recommends that ART is initiated within 7 days following diagnosis and ART initiation on the same‐day in patients who are ready to start [17, 18]. In light of this policy and various country level policies around universal test and treat and same‐day ART, more evidence about the impact of delivering same‐day ART at scale is needed, outside of research settings, to understand treatment outcomes and deliver interventions to improve retention and viral suppression in those patients.

Our study sought to evaluate the impact of same‐day ART on LTFU and mortality when offered at scale in the public health sector in South Africa, comparing with treatment outcomes in patients who initiated after their HIV diagnosis.

## 2 Methods

### 2.1 Data source and study population

We conducted an analysis of a clinic‐based prospective cohort using routinely collected HIV patient level data that was entered into the electronic medical record system, called TIER.Net [19]. We reviewed de‐identified electronic HIV medical records of patients in care in 379 public health facilities, including hospitals, in six high HIV burden districts (Sedibeng in Gauteng Province, Alfred Nzo in Eastern Cape Province, Ugu, King Cetshwayo and Harry Gwala in KwaZulu Natal Province and Gert Sibande in Mpumalanga Province) in South Africa. BroadReach provided support to the HIV treatment programme with the Department of Health with US Government PEPFAR funding. BroadReach is an organization that has worked with the Department of Health in South Africa since 2008.

Before September 2016, patients living with HIV were identified as ART‐eligible according to their CD4 cell count, which had to be <500 cells/µL for non‐pregnant adults. When the Government changed the policy to universal test and treat [20], most clinics started same‐day ART initiation for PLHIV. Some clinics may have delayed the implementation of same‐day ART until they received training on the new guidelines. Once clinicians were trained on the new guidelines, it was up to their discretion about whether or not to initiate a patient with same‐day ART, based on guidelines that included confirmation of “willingness and readiness to start ART.” The guidelines state that if the patient is diagnosed with TB then the clinician should start ART after TB treatment, within eight weeks [2, 20].

We categorized patients according to timing of ART initiation from HIV diagnosis date: same‐day, one to seven, eight to twenty‐one and ≥ twenty‐two days. Patients who initiated ART between September 2016 and May 2018 in six high HIV burden districts and were aged 0 to 85 years were eligible for inclusion. Patients were followed‐up until database closure (31 July 2018). Our analysis was restricted to patients who had an HIV diagnosis date and ART initiation date on file. We excluded patients with previous ART exposure (who may differ from first time ART patients) and patients who had transferred into care from another facility already on ART.

### 2.2 Outcomes

The primary treatment outcome of interest was LTFU was patients who were >90 days from the last clinical visit or drug pick‐up between analysis closure (May 2018) database closure (31 July 2018). We recoded LTFU in the database as it may have been under‐reported in TIER.Net as providers and data capturers had to allocate the patient a LTFU status in the database, whereas we identified many participants who had been out of care for >90 days but did not have a LTFU status allocated to them. For patients who did not return following their same‐day ART initiation, we assigned them one day of follow‐up to ensure they were included in our analyses. The secondary treatment outcome was all‐cause mortality. Mortality reporting varies by site and may be delayed in reporting mortality in the database. We also describe time to VL and elevated VL after ART initiation. Elevated viral VL was defined as having one VL > 1000 copies/mL after ART initiation among patients with ≥3 months on ART (South African Department of Health definition [2]). In South Africa the first VL is done 6‐months after ART start. VL is reported as a proportion of those retained and reported into the database and does not reflect population level VL.

### 2.3 Statistical analysis

Patient‐level baseline characteristics at ART initiation (sex by pregnancy at ART start, age, CD4 count, province of ART initiation and year of ART initiation) and any tuberculosis (TB) treatment status during follow‐up were described using medians and interquartile ranges (IQR) for continuous variables and frequencies and proportions were used for categorical variables.

Time to LTFU and mortality were assessed and compared by timing of ART initiation (same‐day vs. one to seven days, eight to twenty‐one days and ≥twenty‐two days) using Kaplan‐Meier estimators. We investigated predictors of LTFU and mortality using univariate and multivariable Cox proportional hazards models stratified by sex, adjusting for *a priori* characteristics including year of ART start and geography (to control for differences by district and site). The crude and adjusted hazard ratios (HR) with 95% confidence intervals (CI) were reported. We did a sensitivity sub‐group analyses by district to examine if the associations were driven by the large sample size. In these analyses, we excluded districts which had treatment outcome proportions exceeding the overall proportion. For all our models, censoring occurred at the time of LTFU, death, transfer out to another facility or at database closure (July 2018) and contributed time until each respective outcome. Data were cleaned and analysed using Stata version 15.1 (College Station, TX, USA).

### 2.4 Human subjects considerations

Data were retrieved from a de‐identified retrospective analysis of patients’ electronic charts. Names, dates of birth and ID numbers were removed from the dataset before analysis by the Department of Health staff. Participants provided informed consent to undergo HIV testing and counselling and initiate ART as part of the standard of care. University of California Los Angeles’s Institutional Review Board provided exemption (UCLA IRB#19‐000227).

## 3 Results

Overall, 92,609 patients contributed 43,922 person‐years’ of follow‐up after ART initiation, with a median follow‐up time of 246 days (IQR = 112 to 455). Of those patients, 33,399 (36%) had initiated ART on the same‐day as their HIV diagnosis date and had a median follow‐up time of 174 days (IQR = 85 to 349). Patients who initiated ART later had a longer median follow‐up time: one to seven days (n = 18,388 (20%); 239 days (IQR = 112 to 426)), eight to twenty‐one days (n = 14,347 (15%); 285 days (IQR = 131 to 481)) and ≥22 days (n = 26,45 (29%); 371 days (IQR = 161 to 560)).

**Figure 1:**
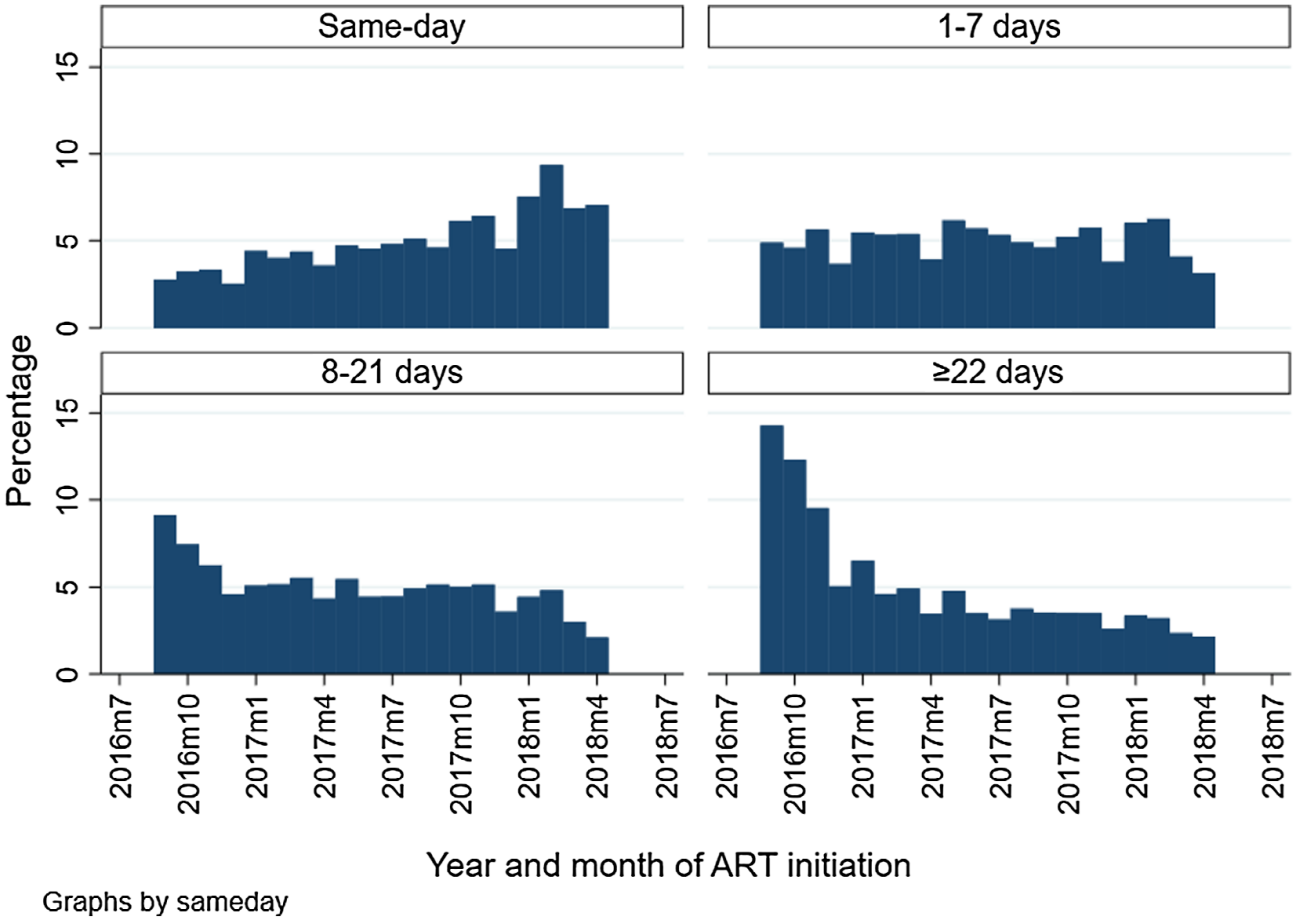
Quarterly rollout of the same‐day ART initiation policy in South Africa from September 2016 to May 2018, by timing of ART inititation. Within each category of timing of ART, the proportion who initiated in that time period is represented. The numerator is the number of paitents who initiated ART in a quarter, and the denominator is the total who initiated ART in the each of the categories (same‐day, one to seven day, eight to twenty‐one day and ≥22 day catergories).

Same‐day patients were predominantly non‐pregnant females (59% same‐day) and aged 25 to 34 years (40% same‐day). Same day initiation occurred in 74% of pregnant females versus 35% of non‐pregnant females. Additionally, infants were the most likely to be initiated same day (56%) followed by those in the 15 to 34 age groups. Only 27% of patients >45 years initiated ART on the day of diagnosis (Table 1). The median CD4 count at ART initiation was similar among same‐day patients (363 cells/µL, IQR = 20 to 561) and one to seven day patients (313 cells/µL, IQR = 158 to 503). The highest proportion of baseline CD4 counts were missing in same‐day patients (37%). Any documented TB treatment during follow‐up was lower for the same‐day and one to seven day patients compared to patients who were initiated >7 days (1.8% same‐day and 1.5% for one to seven day patients). Same day initiation was considerably more prevalent as a proportion of total initiations in the Eastern Cape compared to districts in other provinces; and particularly low in Gauteng (Eastern Cape 68%, KwaZulu Natal 37%, Mpumalanga 24%, Gauteng 17%). Initiation within the first week of diagnosis improved from 2016 to 2018 – more than doubling from 34% to 74% of all initiations. Same day initiations specifically improved from 18% to 54% of all initiations (Figure 1).

**Table 1:** Baseline demographics and health factors of patients initiating ART from September 2016 to May 2018 in six high HIV burden districts of South Africa

	Total (n = 92 609) (246 days, IQR = 112 to 455)	Same‐day ART (n = 33 399) (174 days, IQR = 85 to 349)	1 to 7 days ART (n = 18 388) (239 days, IQR = 112 to 426)	8 to 21 days ART (n = 14 347) (285 days, IQR = 131 to 481)	≥22 days ART (n = 26 495) (371 days, IQR = 161 to 560)
Sex, n (%)					
Non‐pregnant females	53 480 (58.7)	18 856 (56.4)	10 394 (56.6)	7 862 (54.8)	16 368 (62.7)
Pregnant females	6 285 (6.8)	4 638 (13.9)	559 (3.0)	225 (1.6)	863 (3.3)
Male	32 844 (35.5)	9 905 (29.7)	7 435 (40.4)	6 240 (43.6)	9 264 (35.0)
Age (years), n (%)					
Median (IQR)	32.1 (25.8 to 40.6)	30.5 (24.6 to 37.9)	32.6 (26.2 to 41.2)	33.7 (27.1 to 41.2)	33.1 (26.4 to 42.3)
0 to 1	711 (0.8)	400 (1.2)	82 (0.5)	112 (0.8)	117 (0.4)
2 to 14	2098 (2.2)	752 (2.3)	392 (2.1)	304 (2.1)	650 (2.5)
15 to 24	17,650 (19.1)	7713 (23.1)	3293 (17.9)	2154 (15.0)	4490 (17.0)
25 to 34	35,418 (38.2)	13,492 (40.4)	6993 (38.0)	5236 (36.6)	9697 (36.6)
3 to 44	21,355 (23.1)	6819 (20.4)	4429 (24.1)	3743 (26.1)	6364 (24.0)
≥45	15 ,77 (16.6)	4223 (12.6)	3199 (17.4)	2778 (19.4)	5177 (19.5)
CD4 count (cells/µL), n (%)					
Median (IQR)	348 (179 to 560)	363 (204 to 561)	313 (158 to 503)	283 (133 to 376)	418 (210 to 631)
<500	44,732 (48.3)	14,360 (43.0)	10,647 (57.9)	8767 (61.2)	10,958 (41.4)
≥500	20,314 (21.9)	6614 (19.8)	3596 (19.6)	2589 (18.1)	7515 (28.4)
Missing	27,563 (29.8)	12,425 (37.2)	4145 (22.5)	2971 (20.7)	8022 (30.2)
Any TB treatment during follow‐up, n (%)					
Yes	2796 (3.0)	604 (1.8)	280 (1.5)	800 (5.6)	1112 (4.2)
No/unknown	89,813 (97.0)	32,795 (98.2)	18,108 (98.5)	13,527 (94.4)	25,383 (95.8)
Province, n (%)					
KwaZulu‐Natal	33,496 (36.2)	12,512 (37.5)	6044 (32.9)	4308 (30.1)	10,632 (40.1)
Gauteng	20,085 (21.7)	3363 (10.1)	5940 (32.3)	4731 (33.0)	6051 (22.9)
Mpumalanga	20,416 (22.0)	4855 (14.5)	5028 (27.3)	4245 (29.6)	6288 (23.7)
Eastern Cape	18,612 (20.1)	12,669 (37.9)	1376 (7.5)	1043 (7.3)	3524 (13.3)
Year of ART initiation, n (%)					
2016	23,728 (25.6)	4330 (13.0)	3699 (20.1)	4163 (29.1)	11,536 (43.6)
2017	48,218 (52.1)	17,830 (53.4)	10,731 (58.4)	7884 (55.0)	11,773 (44.4)
2018	20,663 (22.3)	11,239 (33.6)	3958 (21.5)	2280 (15.9)	3186 (12.0)

Median follow‐up time for each group is indicated in the column heading.

### 3.1 Loss to follow up (LTFU)

Overall, 30,024 patients (32%) were classified as LTFU, with a median time to LTFU of 112 days (IQR = 1 to 307). Among same‐day patients, 33% (n = 11,114) were classified as LTFU with a median time of 55 days (IQR = 1 to 185). A similar proportion of LTFU noted in the one to seven day patients (n = 5,708, 31%), eight to twenty‐one day patients (n = 4,403, 31%) and ≥22 day patients (n = 8,799 33%). Later initiating patients had a longer median time to LTFU as follows: 113 days (IQR = 19 to 283) for one to seven day patients, 142 days (IQR = 28 to 248) for eight to twenty‐one day patients and 205 days (IQR = 42 to 417) for ≥22 day patients. Among patients with TB, a higher proportion of patients were LTFU in the ≥22 day group (43%) compared to same‐day patients (22%), one to seven day patients (8.8%) and ≥22 day patients (26%).

**Figure 2:**
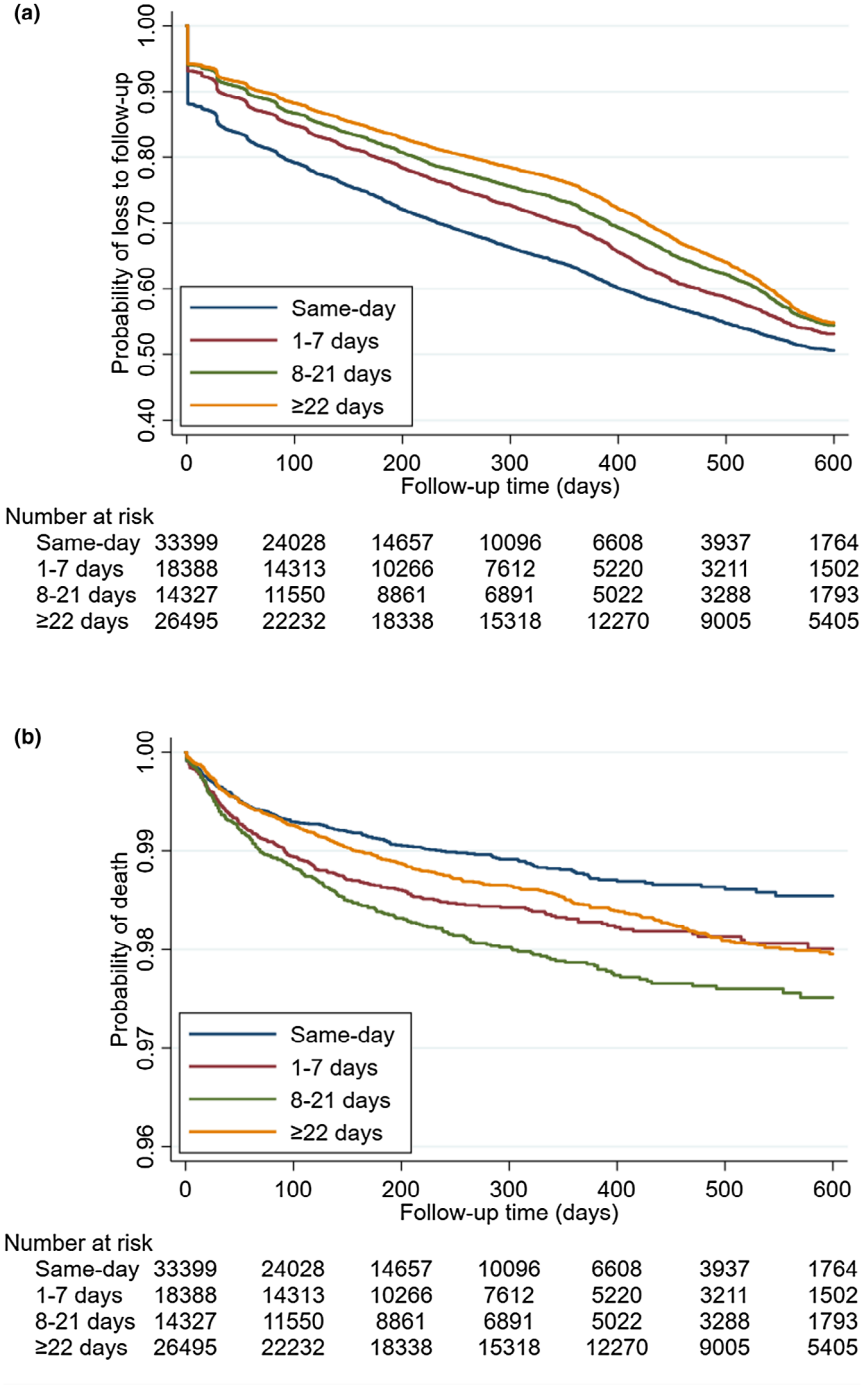
Survival probability according to ART initiation groups (same‐day; one to seven days; 8 to 21 days; ≥22 days) for the outcomes (a) loss to follow‐up (log rank *p* < 0.0001) and (b) mortality (log rank *p* < 0.0001). *Note: the y‐axis scale is different for (a and b).

In the unadjusted Kaplan‐Meier plots, same‐day ART initiation predicted LTFU (Figure 2a) and mortality (Figure 2b). An increased risk of LTFU was noted in same‐day patients versus ≥22 day patients (HR 1.45, 95% CI = 1.41 to 1.49, Table 2), which persisted in the multivariable model (aHR = 1.37, 95% CI = 1.32 to 1.43, Table 2) controlling for sex, age, baseline CD4, TB treatment during follow‐up, province and year of ART initiation. Similar associations for timing of ART initiation persisted when ≥1 day patients were combined. Male sex (aHR = 1.20, 95% CI = 1.16 to 1.23, Table 2, model 1b) and age 15 to 24 years (aHR = 1.75, 95% CI = 1.67 to 1.84, 15 to 24 years vs. ≥45 years) were associated with a higher risk of LTFU, after adjustment. In addition, TB treatment during follow‐up (aHR = 1.19, 95% CI = 1.08 to 1.30), initiating ART in Gauteng Province (aHR = 1.13, 95% CI = 1.09 to 1.18) and in 2017 (aHR = 1.49, 95% CI = 1.44 to 1.55) predicted LTFU after adjustment. When the model was stratified by sex, similar associations persisted for age, timing of ART initiation, province of ART initiation, and year of ART initiation (Table 2, model 2 and 3).

**Table 2:** Crude and adjusted associations for loss to follow‐up (no clinical visits or drug pick up in the 90 days before database closure) and timing of ART initiation among patients who initiated ART from September 2016 to May 2018 by sex

	All		Males		Females	
	Model 1a Univariate HR (95% CI)	Model 1b Multivariable aHR^a^ (95% CI) (n = 65 046)	Model 2a Univariate HR (95% CI)	Model 2b Multivariable aHR^b^ (95% CI) (n = 23 729)	Model 3a Univariate HR (95% CI)	Model 3b Multivariable aHR^b^ (95% CI) (n = 41 317)
Sex						
Male	**1.08 (1.05 to 1.10)**	**1.20 (1.16 to 1.23)**	–	–	–	–
Age at art start (years)						
0 to 1	**2.00 (1.77 to 2.26)**	**1.91 (1.56 to 2.35)**	**1.36 (1.11 to 1.68)**	1.30 (0.91 to 1.86)	**2.60 (2.22 to 3.03)**	**2.51 (1.93 to 3.23)**
2 to 14	0.76 (0.68 to 0.84)	**0.63 (0.54 to 0.72)**	**0.67 (0.58 to 0.77)**	**0.54 (0.44 to 0.67)**	**0.83 (0.72 to 0.95)**	**0.72 (0.59 to 0.87)**
15 to 24	**1.78 (1.71 to 1.85)**	**1.75 (1.67 to 1.84)**	**1.67 (1.54 to 1.80)**	**1.57 (1.43 to 1.72)**	**1.93 (1.84 to 2.03)**	**1.80 (1.70 to 1.92)**
25 to 34	**1.47 (1.42 to 1.53)**	**1.42 (1.36 to 1.48)**	**1.45 (1.37 to 1.53)**	**1.42 (1.33 to 1.51)**	**1.52 (1.45 to 1.59)**	**1.41 (1.33 to 1.50)**
35 to 44	**1.22 (1.17 to 1.27)**	**1.14 (1.09 to 1.20)**	**1.17 (1.37 to 1.53)**	**1.10 (1.03 to 1.18)**	**1.23 (1.17 to 1.30)**	**1.17 (1.10 to 1.25)**
≥45	1.0	1.0	1.0	1.0	1.0	1.0
Time to ART start						
Same‐day ART	**1.45 (1.41 to 1.49)**	**1.37 (1.32 to 1.43)**	**1.35 (1.28 to 1.42)**	**1.36 (1.27 to 1.46)**	**1.52 (1.46 to 1.57)**	**1.35 (1.28 to 1.42)**
1 to 7 days ART	**1.17 (0.13 to 1.21)**	**1.13 (1.09 to 1.18)**	**1.10 (1.05 to 1.16)**	**1.12 (1.05 to 1.19)**	**1.20 (1.15 to 1.25)**	**1.14 (1.08 to 1.21)**
8 to 21 days ART	**1.05 (1.02 to 1.09)**	**1.06 (1.02 to 1.11)**	1.01 (0.96 to 1.07)	1.05 (0.98 to 1.13)	**1.06 (1.01 to 1.11)**	**1.07 (1.01 to 1.13)**
≥22 days ART	1.0	1.0	1.0	1.0	1.0	1.0
Same‐day ART	**1.37 (1.34 to 1.40)**	**1.28 (1.24 to 1.33)**	**1.30 (1.25 to 1.36)**	**1.29 (1.22 to 1.37)**	**1.42 (1.38 to 1.46)**	**1.27 (1.22 to 1.33)**
≥1 days ART	1.0	1.0	1.0	1.0	1.0	1.0
Baseline CD4 count (cells/µL)						
<500	**1.11 (1.08 to 1.15)**	1.03 (1.00 to 1.07)	**1.11 (1.05 to 1.17)**	1.05 (0.99 to 1.11)	**1.09 (1.05 to 1.13)**	1.02 (0.98 to 1.06)
≥500	1.0	1.0	1.0	1.0	1.0	1.0
Any TB treatment during follow‐up						
Yes	**1.22 (1.13 to 1.31)**	**1.19 (1.08 to 1.30)**	**1.19 (1.09 to 1.31)**	**1.20 (1.07 to 1.35)**	**1.18 (1.04 to 1.33)**	1.16 (0.99 to 1.06)
No	1.0	1.0	1.0	1.0	1.0	1.0
Province						
KwaZulu‐Natal	1.0	1.0	1.0	1.0	1.0	1.0
Gauteng	**0.89 (0.86 to 0.91)**	**1.13 (1.09 to 1.18)**	**0.90 (0.86 to 0.95)**	**1.16 (1.09 to 1.23)**	**0.87 (0.84 to 0.91)**	**1.11 (1.06 to 1.17)**
Mpumalanga	**0.81 (0.79 to 0.84)**	0.98 (0.95 to 1.02)	**0.84 (0.80 to 0.88)**	1.04 (0.98 to 1.10)	**0.79 (0.76 to 0.91)**	**0.95 (0.90 to 0.99)**
Eastern Cape	**0.94 (0.91 to 0.97)**	**0.92 (0.87 to 0.96)**	0.95 (0.89 to 1.00)	0.95 (0.88 to 1.03)	**0.94 (0.91 to 0.98)**	**0.90 (0.86 to 0.95)**
Year of ART initiation						
2016	1.0	1.0	1.0	1.0	1.0	1.0
2017	**1.47 (1.43 to 1.51)**	**1.49 (1.44 to 1.55)**	**1.30 (1.24 to 1.36)**	**1.33 (1.25 to 1.41)**	**1.55 (1.50 to 1.61)**	**1.60 (1.53 to 1.68)**
2018	**1.60 (1.54 to 1.67)**	**1.40 (1.33 to 1.48)**	**1.40 (1.31 to 1.50)**	**1.27 (1.17 to 1.38)**	**1.72 (1.63 to 1.81)**	**1.49 (1.39 to 1.59)**
Pregnant at ART start						
Yes	**–**	**–**	–	–	**1.41 (1.35 to 1.47)**	**1.08 (1.02 to 1.15)**

Bold values *p* < .05.

^a^ Adjusted for sex, age, time to ART start (same‐day, one to seven days, eight to twenty‐one days and ≥twenty‐two days), baseline CD4 count, TB treatment during follow‐up, province, year of ART initiation, pregnant at ART start

^b^ Adjusted for adjusted for sex, age, time to ART start (same‐day, one to seven days, eight to twenty‐one days and ≥twenty‐two days), baseline CD4 count, TB treatment during follow‐up, province, year of ART initiation, pregnant at ART start.

### 3.2 Mortality

All‐cause mortality was slightly lower in same‐day patients (n = 285, 0.9%) compared to one to seven day patients (n = 250, 1.4%), eight to twenty‐one day patients (n = 253, 1.8%) and ≥twenty‐two day patients (n = 381, 1.4%). Although median time to mortality was shorter among same‐day patients (51 days, IQR = 16 to 141) compared to ≥twenty‐two day patients (112 days, IQR = 34 to 255). Among patients with TB, a higher proportion of patients who initiated ART ≥twenty‐two days died (45%) compared to same‐day patients (15%), one to seven day patients (15%) and ≥twenty‐two day patients (25%).

In the univariate analysis of mortality, same‐day initiation was protective (HR = 0.78, 95% CI = 0.67 to 0.91, same‐day vs. ≥twenty‐two day, Table 3, model 1) of all‐cause mortality. After adjustment of sex, age, baseline CD4, TB treatment, province of ART initiation and year of ART initiation this association was no longer predictive (aHR = 0.82, 95% CI 0.65 to 1.01, same‐day vs. ≥twenty‐two day, Table 3, model 1b). Similar associations for timing of ART initiation persisted when ≥1 day patients were combined. The risk of all‐cause mortality was higher in patients who: were male (aHR = 1.70, 95% CI 1.47 to 1.96), had a lower CD4 count (aHR = 3.13, 95% CI 2.49 to 3.93 for <500 vs. ≥500 copies/mL) and had any TB treatment during follow‐up (aHR = 6.64, 95% CI 5.52 to 8.22), after adjustment (Table 3, model 1b). Initiating ART in the Eastern Cape increased the risk of mortality (aHR 1.28, IQR = 1.02 to 1.61), after adjustment. Patients who initiated ART in 2018 were less likely to die (aHR = 0.66, 95% CI 0.51 to 0.86, 2018 vs. 2016). When the model was stratified by sex, similar associations persisted for age, timing of ART initiation, province of ART initiation and year of ART initiation (Table 3, model 2 and 3). In our sub‐group analyses, similar associations for timing of ART initiation were observed for all models (Tables S1 and S2).

**Table 3:** Crude and adjusted associations for mortality and timing of ART initiation among patients who initiated ART from September 2016 to May 2018 by sex

	All		Males		Females	
	Model 1a Univariate HR (95% CI)	Model 1b Multivariable aHR^a^ (95% CI) (n = 65 046)	Model 2a Univariate HR (95% CI)	Model 2b Multivariable aHR^b^ (95% CI) (n = 23 729)	Model 3a Univariate HR (95% CI)	Model 3b Multivariable aHR^b^ (95% CI) (n = 41 317)
Sex						
Male	**2.57 (2.28 to 2.88)**	**1.70 (1.47 to 1.96)**	–	–	–	–
Age at art start (years)						
0 to 1	0.81 (0.44 to 1.47)	0.59 (0.15 to 2.36)	0.67 (0.30 to 1.51)	0.95 (0.23 to 3.85)	0.97 (0.40 to 2.37)	–
2to 14	**0.29 (0.18 to 0.48)**	**0.14 (0.05 to 0.38)**	**0.24 (0.12 to 0.47)**	**0.06 (0.01 to 0.41)**	**0.34 (0.16 to 0.73)**	**0.26 (0.08 to 0.82)**
15 to 24	**0.20 (0.16 to 0.25)**	**0.26 (0.19 to 0.36)**	**0.30 (0.20 to 0.46)**	**0.36 (0.22 to 0.58)**	**0.23 (0.17 to 0.32)**	**0.25 (0.16 to 0.37)**
25 to 34	**0.41 (0.36 to 0.48)**	**0.45 (0.38 to 0.54)**	**0.46 (0.68 to 0.56)**	**0.47 (0.37 to 0.60)**	**0.41 (0.33 to 0.51)**	**0.45 (0.35 to 0.59)**
35 to 44	**0.64 (0.55 to 0.74)**	**0.62 (0.52 to 0.74)**	**0.61 (0.51 to 0.74)**	**0.66 (0.53 to 0.82)**	**0.61 (0.48 to 0.77)**	**0.58 (0.44 to 1.77)**
≥45	1.0	1.0	1.0	1.0	1.0	1.0
Time to ART start						
Same‐day ART	**0.78 (0.67 to 0.91)**	0.82 (0.65 to 1.01)	**0.81 (0.65 to 0.99)**	0.82 (0.61 to 1.08)	**0.83 (0.66 to 1.05)**	**0.95 (0.69 to 1.31)**
1 to 7 days ART	1.10 (0.93 to 1.29)	1.03 (0.85 to 1.25)	0.90 (0.73 to 1.12)	0.91 (0.71 to 1.17)	1.23 (0.97 to 1.58)	1.22 (0.91 to 1.63)
8 to 21 days ART	**1.33 (1.14 to 1.56)**	0.99 (0.82 to 1.20)	1.09 (0.89 to 1.34)	0.95 (0.75 to 1.22)	**1.44 (1.12 to 1.85)**	1.02 (0.75 to 1.38)
≥22 days ART	1.0	1.0	1.0	1.0	1.0	1.0
Same‐day ART	**0.70 (0.61 to 0.80)**	0.87 (0.72 to 1.05)	**0.81 (0.67 to 0.97)**	0.85 (0.66 to 1.10)	**0.72 (0.59 to 0.88)**	0.89 (0.66 to 1.18)
≥1 days ART	1.0	1.0	1.0	1.0	1.0	1.0
Baseline CD4 count (cells/µL)						
<500	**4.08 (3.27 to 5.09)**	**3.13 (2.49 to 3.93)**	**2.88 (2.09 to 3.97)**	**2.54 (1.84 to 3.51)**	**4.10 (3.01 to 5.57)**	**3.57 (2.60 to 4.89)**
≥500	1.0	1.0	1.0	1.0	1.0	1.0
Any TB treatment during follow‐up						
Yes	**8.17 (6.97 to 9.57)**	**6.64 (5.52 to 8.22)**	**5.06 (4.14 to 6.17)**	**5.30 (4.13 to 6.81)**	**11.64 (8.96 to 15.14)**	**10.52 (7.63 to 14.51)**
No	1.0	1.0	1.0	1.0	1.0	1.0
Province						
KwaZulu‐Natal	1.0	1.0	1.0	1.0	1.0	1.0
Gauteng	**1.19 (1.02 to 1.38)**	1.09 (0.91 to 1.31)	0.99 (0.81 to 1.21)	1.02 (0.80 to 1.30)	**1.34 (1.06 to 1.69)**	1.23 (0.93 to 1.65)
Mpumalanga	0.97 (0.83 to 1.14)	1.07 (0.88 to 1.29)	0.87 (0.71 to 1.07)	1.06 (0.83 to 1.36)	1.02 (0.79 to 131)	1.09 (0.80 to 1.47)
Eastern Cape	1.16 (0.99 to 1.35)	**1.28 (1.02 to 1.61)**	**1.27 (1.02 to 1.57)**	1.35 (0.99 to 1.83)	1.21 (0.95 to 1.53)	1.18 (0.84 to 1.65)
Year of ART initiation						
2016	1.0	1.0	1.0	1.0	1.0	1.0
2017	**1.24 (1.08 to 1.41)**	1.07 (0.90 to 1.25)	1.19 (0.99 to 1.42)	1.05 (0.85 to 1.31)	1.18 (0.97 to 1.44)	1.06 (0.83 to 1.37)
2018	0.90 (0.74 to 1.11)	**0.66 (0.51 to 0.86)**	0.93 (0.72 to 1.21)	**0.70 (0.50 to 0.97)**	0.76 (0.55 to 1.05)	**0.62 (0.40 to 0.94)**
Pregnant at ART start						
Yes	**–**	**–**	–	–	**0.22 (0.12 to 0.40)**	**0.09 (0.02 to 0.37)**

Bold values *p* < .05.

^a^ Adjusted for sex, age, time to ART start (same‐day, one to seven days, eight to twenty‐one days and ≥twenty‐two days), baseline CD4 count, TB treatment during follow‐up, province, year of ART initiation, pregnant at ART start

^b^ adjusted for adjusted for sex, age, time to ART start (same‐day, one to seven days, eight to twenty‐one days and ≥twenty‐two days), baseline CD4 count, TB treatment during follow‐up, province, year of ART initiation, pregnant at ART start.

### 3.3 Viraemia

In this cohort of patients, excluding patients with the outcome LTFU and mortality, 37,660 patients (61%) had a VL result on file during follow‐up and 23,756 patients had a missing VL (39%). Missing VLs were as follows: 50% in same‐day patients, 62% in one to seven day patients, 67% in eight twenty‐one day patients and 73% in ≥twenty‐two day patients. The median time to the first VL test was 221 days (IQR 170 to 364), with VL testing occurring sooner in same‐day patients (195 days, IQR = 154 to 353 days). First documented VL tests occurred later in patients who initiated ART later: 201 days (IQR = 169 to 358) in one to seven day patients, 217 days (IQR = 174 to 364) in eight to twenty‐one day patients, and 339 days (IQR = 186 to 373) ≥twenty‐two day patients. Among patients with a VL test (n = 49,020), 3,746 (10%) had an elevated VL (>1000 copies/mL) of which the highest proportion was observed for same‐day patients (14%). Later initiating patients experienced an elevated VL as follows: 8.7% for one to seven day patients, 9.5% for eight to twenty‐one day patients and 7.7% for ≥ twenty‐two day patients (Data not tabled).

## 4 Discussion

Our study is one of the first studies to evaluate the association between same‐day ART and treatment outcomes at scale in South African public health facilities [21]. The South African Government launched a policy of universal test and treat, including same‐day ART, in September 2016 [2]. Patients who initiated ART on the same‐day as their diagnosis were predominantly female and younger. The proportion of patients who initiated same‐day increased from 3% in September 2016 to 7% in April 2018. Our study found that same‐day ART patients had increased risk of being LTFU, including rapid LTFU compared with patients who initiated ART after their date of diagnosis. Mortality was slightly lower in same‐day ART patients.

Prior randomized control trials on the impact of same‐day ART on treatment outcomes demonstrated lower LTFU and higher viral suppression when comparing same‐day patients to the standard of care [9, 10, 11, 15]. Those studies used additional resources such as point of care CD4 monitoring, additional training and support provided to clinicians to improve the clinical screening, and x‐rays for TB prior to entering the study. Prior observational studies on same‐day ART were in pregnant women only or did not have a comparison group [15, 22]. Our study differs because we evaluated the effect of same‐day ART vs. ≥1 day ART initiation as the standard of care in over 350 South African public health facilities. Similar to our study, a recent South African clinical file review in Gauteng and Limpopo Provinces found that same day ART was associated with poorer retention [16]. During the time period of our study, BroadReach provided additional human resource support including additional nurses, training for nurses to initiate ART and clinical mentorship, as well as data capturing and cleaning.

The risk of LTFU and mortality was most marked in men who had a 20% increased risk of LTFU and a 62% increased risk of mortality compared to female patients. Prior research has demonstrated that South African men have suboptimal levels of HIV testing, ART initiation, retention and survival on treatment and have poorer HIV outcomes than their female counterparts, suggesting that these men remain underserved in the context of the HIV response [5, 6, 23, 24, 25]. Interventions are urgently required to address the discrepancy in HIV care in South African men, including differentiated care that will reduce the barriers to accessing regular ART.

Overall, we demonstrate that approximately one‐third of patients were LTFU and that LTFU is faster in same‐day ART patients. These findings are in line with another recent South African study [16]. In addition, our study found that same‐day patients received VL testing earlier than non‐same day patients (192 days vs. 335 days in ≥twenty‐two day patients). However, in patients with a VL test, 16% of same day patients were viraemic at their first VL, compared to 9% for ≥twenty‐two day patients. However, the difference in viraemia may be explained by the time difference in receiving a VL test in those groups.

There is a possibility that without additional counselling, adherence support and interventions to improve adherence and retention, including differentiated care, the risks of same‐day ART may outweigh the benefits in terms of retention and viral suppression over time [7]. Considering our findings, there is a need for improved, adapted counselling to improve retention and viral suppression in patients who start ART, are healthy and may have limited pre‐ART initiation counselling. Furthermore, differentiated care that adapts HIV services to reflect the needs of various PLHIV, should be expanded as some patients will not need additional interventions and others will have risk factors that can be addressed through differentiated care, including community based ART delivery [26]. Additionally, patients newly diagnosed may need time to discuss their HIV status with sex partners, family and friends who may in turn ensure that the patient is adherent on ART [27]. Importantly, the benefits of same‐day ART are diminished if adherence and retention remain poor, and the risk of development of resistant HIV strains may increase [15]; though this risk should diminish with rollout of dolutegravir‐based maintenance therapy [28]. We recommend that improved education and counseling be integrated into the standard of care to ensure that healthy patients appreciate the advantages of early ART for their health and survival and that those who have an undetectable viral load cannot transmit the virus to sexual partners or through giving birth (U=U). We also recommend that interventions target those at highest risk of LTFU and viraemia including adult men, young patients and those with low baseline CD4 cell counts or TB infection.

In South Africa, clinicians are trained to conduct TB screening and screening for signs and symptoms of cryptococcal meningitis prior to ART initiation. However, our study found that 43% of same‐day ART patients were initiated with low CD4 cell counts (<500 cells/µL), and 37% had missing CD4 results (higher than later ART start groups). In addition, 2% received TB treatment during follow‐up (though we do not know if they were on TB treatment at ART start). Despite this inclusion of potentially symptomatic and immuno‐compromised patients, we did not see an increased mortality in the same‐day ART group. We strongly recommend that the Department of Health and partners improve the training of clinicians to ensure that they conduct improved clinical screening, including TB screening, to avoid same‐day initiation of patients with low CD4 count who may be at risk of IRIS. Clinicians should defer rapid ART start for any patients that have clinical symptoms suggestive of TB or cryptococcal meningitis [^29^].

Our study has several limitations. First, we did not collect additional clinical, demographic or behavioural data but used routinely collected data in patient clinical files. Because of the nature of using electronic patient records, our results may over or under‐estimate the true effect of same‐day ART because of missing and incomplete data. Second, mortality reporting varies by site and may be delayed in reporting mortality in the database. Mortality may be under‐reported if the healthcare worker is unable to contact the family and get verbal confirmation. Furthermore, the lack of a statistical difference in mortality between the two groups could be due to the low overall proportion of deaths (1%) and limited power. Third, there may be silent transfers as well who were classified as LTFU but may be in care in another facility. Finally, we could not determine the rate of confirmed virologic failure due to the limited follow‐up time.

## 5 CONCLUSIONS

Our findings suggest that same‐day ART is being offered in South African public health facilities, and that the proportion of patients being offered same‐day ART has increased between 2016 and 2018. Same‐day ART patients had increased risk of LTFU, but lower overall mortality when compared to patients who started ART after their diagnosis. Men had highest risk of mortality and LTFU. Patients may require additional counselling and differentiated care to improve adherence and retention to achieve viral suppression. Implementation science studies are urgently needed to evaluate how best to start ART quickly in patients who are ready, but also improve retention and viral suppression in those patients.

## Competing Interests

The authors have no funding or conflicts of interest to disclose.

## Authors’ Contributions

DJD led the data collection, analysis and wrote the first draft of the manuscript. KK led the updated data analysis, edited the methods section and results sections of the paper and reviewed the overall paper for accuracy. CS assisted with data collection, data quality, discussion section, and reviewed and revised the manuscripts and approved the final submission. MP assisted with data collection, data quality, discussion section, and reviewed and revised the manuscripts and approved the final submission. NM assisted with data collection, data quality, discussion section, and reviewed and revised the manuscripts and approved the final submission. KH assisted with data collection, data quality, discussion section, and reviewed and revised the manuscripts and approved the final submission. SS assisted with data collection, data quality, discussion section, and reviewed and revised the manuscripts and approved the final submission. TM reviewed the study concept, reviewed and revised the manuscripts and approved the final submission.

## Supporting information


**Table S1.** Crude and adjusted associations for loss to follow‐up (no clinical visits or drug pick up in the 90 days before database closure) and timing of ART initiation among patients who initiated ART from September 2016 to May 2018 by sex. Districts that had >32.4% LTFU were excluded for this analysis
**Table S2.** Crude and adjusted associations for mortality and timing of ART initiation among patients who initiated ART from September 2016 to May 2018 by sex. Districts that had >1.2% mortality were excluded for this analysis
