# Supplementary material for: Same‐day antiretroviral therapy is associated with increased loss to follow‐up in South African public health facilities: a prospective cohort study of patients diagnosed with HIV
Source: J Int AIDS Soc. 2020 Jun 8;23(6):e25529. doi: 10.1002/jia2.25529 (PMC7277782; doi:10.1002/jia2.25529)
Supplement: Supplementary file 1 — Table S1. Crude and adjusted associations for loss to follow‐up (no clinical visits or drug pick up in the 90 days before database closure) and timing of ART initiation among patients who initiated ART from September 2016 to May 2018 by sex. Districts that had >32.4% LTFU were excluded for this analysis Table S2. Crude and adjusted associations for mortality and timing of ART initiation among patients who initiated ART from September 2016 to May 2018 by sex. Districts that had >1.2% mortality were excluded for this analysis [file JIA2-23-e25529-s001.docx]

**Supplementary Table 1.** Crude and adjusted associations for loss to follow-up (no clinical visits or drug pick up in the 90 days before database closure) and timing of ART initiation among patients who initiated ART from September 2016 to May 2018 by sex. Districts that had >32.4% LTFU were excluded for this analysis.

|  | **All** | | **Males** | | **Females** | |
| --- | --- | --- | --- | --- | --- | --- |
|  | **Model 1a** Univariate HR (95% CI) | **Model 1b** Multivariable aHR* (95% CI)  (n =43 721) | **Model 2a** Univariate HR (95% CI) | **Model 2b** Multivariable aHRˠ (95% CI)  (n =15 551) | **Model 3a** Univariate HR (95% CI) | **Model 3b** Multivariable aHRˠ (95% CI)  (n =28 170) |
| **Sex** |  |  |  |  |  |  |
| Male | **1.11 (1.07-1.14)** | **1.26 (1.20-1.31)** | - | - | - | - |
| **Age at art start (years)** |  |  |  |  |  |  |
| 0-1 | **1.97 (1.67-2.34)** | **1.78 (1.32-2.40)** | 1.34 (1.00-1.79) | 1.28 (0.77-2.15) | **2.53 (2.05-3.12)** | **2.22 (1.54-3.21)** |
| 2-14 | **0.77 (0.67-0.88)** | **0.60 (0.49-0.73)** | **0.67 (0.55-0.83)** | **0.46 (0.34-0.63)** | 0.85 (0.71-1.01) | **0.75 (0.58-0.96)** |
| 15-24 | **1.80 (1.70-1.89)** | **1.84 (1.72-1.96)** | **1.71 (1.54-1.90)** | **1.62 (1.43-1.84)** | **1.93 (1.81-2.06)** | **1.91 (1.76-2.07)** |
| 25-34 | **1.44 (1.38-1.52)** | **1.44 (1.36-1.52)** | **1.50 (1.39-1.62)** | **1.46 (1.34-1.60)** | **1.44 (1.35-1.53)** | **1.42 (1.32-1.54)** |
| 35-44 | **1.18 (1.12-1.24)** | **1.15 (1.07-1.22)** | **1.17 (1.08-1.26)** | 1.10 (1.00-1.21) | **1.16 (1.08-1.25)** | **1.19 (1.09-1.29)** |
| ≥45 | 1.0 | 1.0 | 1.0 | 1.0 | 1.0 | 1.0 |
| **Time to ART start** |  |  |  |  |  |  |
| Same-day ART | **1.43 (1.38-1.48)** | **1.21 (1.15-1.28)** | **1.31 (1.23-1.40)** | **1.21 (1.10-1.33)** | **1.50 (1.43-1.57)** | **1.21 (1.13-1.30)** |
| 1-7 days ART | 1.01 (0.96-1.06) | 0.96 (0.91-1.02) | 0.93 (0.86-1.00) | 0.92 (0.84-1.01) | 1.04 (0.98-1.11) | 0.99 (0.92-1.07) |
| 8-21 days ART | 0.96 (0.91-1.01) | 0.94 (0.89-1.00) | **0.91 (0.85-0.99)** | 0.94 (0.86-1.03) | 0.96 (0.90-1.02) | 0.94 (0.87-1.02) |
| ≥22 days ART | 1.0 | 1.0 | 1.0 | 1.0 | 1.0 | 1.0 |
|  |  |  |  |  |  |  |
| Same-day ART | **1.44 (1.39-1.49)** | **1.24 (1.18-1.31)** | **1.37 (1.30-1.45)** | **1.26 (1.16-1.36)** | **1.50 (1.44-1.56)** | **1.23 (1.16-1.31)** |
| ≥1 days ART | 1.0 | 1.0 | 1.0 | 1.0 | 1.0 | 1.0 |
| **Baseline CD4 count (cells/µl)** |  |  |  |  |  |  |
| <500 | **1.07 (1.02-1.11)** | 1.02 (0.97-1.52) | 1.08 (1.00-1.17) | 1.06 (0.98-1.14) | 1.03 (0.98-1.08) | 1.00 (0.95-1.05) |
| ≥500 | 1.0 | 1.0 | 1.0 | 1.0 | 1.0 | 1.0 |
| **Any TB treatment during follow-up** |  |  |  |  |  |  |
| Yes | **1.38 (1.24-1.54)** | **1.32 (1.14-1.52)** | **1.31 (1.14-1.50)** | **1.28 (1.07-1.54)** | **1.39 (1.16-1.66)** | **1.37 (1.09-1.73)** |
| No | 1.0 | 1.0 | 1.0 | 1.0 | 1.0 | 1.0 |
| **Province** |  |  |  |  |  |  |
| KwaZulu-Natal | 1.0 | 1.0 | 1.0 | 1.0 | 1.0 | 1.0 |
| Gauteng | **0.94 (0.90-0.98)** | **1.12 (1.06-1.18)** | 0.95 (0.88-1.03) | **1.14 (1.04-1.24)** | 0.92 (0.86-0.97) | 1.12 (1.04-1.19) |
| Mpumalanga | 1.01 (0.97-1.06) | **1.08 (1.03-1.14)** | 1.03 (0.96-1.10) | **1.13 (1.03-1.23)** | 0.99 (0.94-1.05) | 1.05 (0.98-1.13) |
| Eastern Cape | **1.34 (1.29-1.40)** | **1.21 (1.14-1.28)** | **1.29 (1.20-1.39)** | **1.23 (1.11-1.36)** | **1.38 (1.31-1.45)** | **1.21 (1.12-1.29)** |
| **Year of ART initiation** |  |  |  |  |  |  |
| 2016 | 1.0 | 1.0 | 1.0 | 1.0 | 1.0 | 1.0 |
| 2017 | **1.19 (1.15-1.24)** | **1.13 (1.08-1.18)** | 1.04 (0.98-1.11) | 1.02 (0.95-1.10) | **1.28 (1.22-1.34)** | **1.20 (1.13-1.27)** |
| 2018 | **1.64 (1.56-1.73)** | **1.40 (1.31-1.50)** | **1.41 (1.30-1.53)** | **1.25(1.12-1.39)** | **1.78 (1.67-1.34)** | **1.50 (1.37-1.63)** |
| **Pregnant at ART start** |  |  |  |  |  |  |
| Yes | **-** | - | - | - | **1.26 (1.18-1.35)** | 0.98 (0.90-1.07) |

* adjusted for sex, age, time to ART start (same-day, 1-7 days, 8-21 days and ≥22 days), baseline CD4 count, TB treatment during follow-up, province, year of ART initiation, pregnant at ART start

ˠ adjusted for adjusted for sex, age, time to ART start (same-day, 1-7 days, 8-21 days and ≥22 days), baseline CD4 count, TB treatment during follow-up, province, year of ART initiation, pregnant at ART start

**Supplementary Table 2.** Crude and adjusted associations for mortality and timing of ART initiation among patients who initiated ART from September 2016 to May 2018 by sex. Districts that had >1.2% mortality were excluded for this analysis.

|  | **All** | | **Males** | | **Females** | |
| --- | --- | --- | --- | --- | --- | --- |
|  | **Model 1a** Univariate HR (95% CI) | **Model 1b** Multivariable aHR* (95% CI)  (n =31 768) | **Model 2a** Univariate HR (95% CI) | **Model 2b** Multivariable aHRˠ (95% CI)  (n =12 108) | **Model 3a** Univariate HR (95% CI) | **Model 3b** Multivariable aHRˠ (95% CI)  (n =19 660) |
| **Sex** |  |  |  |  |  |  |
| Male | **2.40 (1.97-2.93)** | **1.66 (1.30-2.10)** | - | - | - | - |
| **Age at art start (years)** |  |  |  |  |  |  |
| 0-1 | 0.72 (0.18-2.89) | - | - | - | 1.84 (0.45-7.50) | - |
| 2-14 | **0.22 (0.08-0.60)** | **0.19 (0.05-0.77)** | **0.09 (0.01-0.66)** | - | 0.39 (0.12-1.26) | 0.43 (0.10-1.78) |
| 15-24 | **0.18 (0.11-0.28)** | **0.15 (0.07-0.27)** | **0.36 (0.18-0.72)** | **0.25 (0.10-0.62)** | **0.16 (0.09-0.29)** | **0.10 (0.04-0.25)** |
| 25-34 | **0.39 (0.31-0.51)** | **0.41 (0.31-0.54)** | **0.48 (0.34-0.66)** | **0.48 (0.33-0.70)** | **0.34 (0.23-0.49)** | **0.34 (0.22-0.53)** |
| 35-44 | **0.56 (0.43-.72)** | **0.54 (0.40-0.71)** | **0.58 (0.42-0.81)** | **0.59 (0.41-0.85)** | **0.45 (0.30-0.68)** | **0.48 (0.30-0.75)** |
| ≥45 | 1.0 | 1.0 | 1.0 | 1.0 | 1.0 | 1.0 |
| **Time to ART start** |  |  |  |  |  |  |
| Same-day ART | **0.49 (0.35-0.68)** | 0.68 (0.45-1.01) | **0.47 (0.30-0.74)** | 0.60 (0.35-1.02) | **0.57 (0.35-0.92)** | 0.93 (0.50-1.71) |
| 1-7 days ART | 0.87 (0.67-1.15) | 0.86 (0.63-1.17) | **0.64 (0.44-0.92)** | 0.69 (0.46-1.04) | 1.17 (0.78-1.74) | 1.17 (0.73-1.87) |
| 8-21 days ART | 1.20 (0.93-1.54) | 0.84 (0.63-1.13) | 0.94 (0.68-1.30) | 0.76 (0.52-1.10) | 1.37 (0.93-2.04) | 0.96 (0.60-1.54) |
| ≥22 days ART | 1.0 | 1.0 | 1.0 | 1.0 | 1.0 | 1.0 |
|  |  |  |  |  |  |  |
| Same-day ART | **0.48 (0.35-0.66)** | 0.78 (0.54-1.15) | **0.53 (0.34-0.82)** | 0.71 (0.42-1.18) | **0.51 (0.32-0.80)** | 0.90 (0.51-1.59) |
| ≥1 days ART | 1.0 | 1.0 | 1.0 | 1.0 | 1.0 | 1.0 |
| **Baseline CD4 count (cells/µl)** |  |  |  |  |  |  |
| <500 | **3.19 (2.30-3.44)** | **2.76 (1.95-3.88)** | **2.63 (1.59-4.34)** | **2.55 (1.53-4.24)** | **2.83 (1.81-4.42)** | **2.71 (1.70-4.32)** |
| ≥500 | 1.0 | 1.0 | 1.0 | 1.0 | 1.0 | 1.0 |
| **Any TB treatment during follow-up** |  |  |  |  |  |  |
| Yes | **8.04 (6.05-10.69)** | **9.22 (6.54-12.99)** | **4.84 (4.36-6.98)** | **6.33 (4.05-9.89)** | **12.56 (7.99-19.74)** | **17.56 (10.24-29.08)** |
| No | 1.0 | 1.0 | 1.0 | 1.0 | 1.0 | 1.0 |
| **Province** |  |  |  |  |  |  |
| KwaZulu-Natal | 1.0 | 1.0 | 1.0 | 1.0 | 1.0 | 1.0 |
| Gauteng | 1.21 (0.94-1.56) | 1.15 (0.84-1.56) | 0.93 (0.66-1.31) | 0.96 (0.64-1.44) | **1.51 (1.03-2.20)** | 1.48 (0.92-2.39) |
| Mpumalanga | **1.42 (1.12-1.80)** | **1.56 (1.16-2.11)** | 1.26 (0.93-1.72) | 1.46 (1.00-2.14) | **1.49 (1.03-2.16)** | **1.70 (1.06-2.73)** |
| Eastern Cape | - | - | - | - | - | - |
| **Year of ART initiation** |  |  |  |  |  |  |
| 2016 | 1.0 | 1.0 | 1.0 | 1.0 | 1.0 | 1.0 |
| 2017 | **0.79 (0.63-0.98)** | **0.68 (0.52-0.88)** | 0.78 (0.58-1.05) | 0.72 (0.51-1.00) | **0.71 (0.51-0.99)** | **0.61 (0.41-0.91)** |
| 2018 | **0.71 (0.51-0.98)** | **0.42 (0.28-0.63)** | 0.81 (0.54-1.21) | **0.50 (0.30-0.83)** | **0.48 (0.27-0.85)** | **0.35 (0.18-0.68)** |
| **Pregnant at ART start** |  |  |  |  |  |  |
| Yes | **-** | - | - | - | **0.26 (0.10-0.70)** | 0.36 (0.09-1.49) |

* adjusted for sex, age, time to ART start (same-day, 1-7 days, 8-21 days and ≥22 days), baseline CD4 count, TB treatment during follow-up, province, year of ART initiation, pregnant at ART start

ˠ adjusted for adjusted for sex, age, time to ART start (same-day, 1-7 days, 8-21 days and ≥22 days), baseline CD4 count, TB treatment during follow-up, province, year of ART initiation, pregnant at ART start
